# Evaluation of humoral immune status in porcine epidemic diarrhea virus (PEDV) infected sows under field conditions

**DOI:** 10.1186/s13567-015-0285-x

**Published:** 2015-12-14

**Authors:** Kang Ouyang, Duan-Liang Shyu, Santosh Dhakal, Jagadish Hiremath, Basavaraj Binjawadagi, Yashavanth S. Lakshmanappa, Rui Guo, Russell Ransburgh, Kathryn M. Bondra, Phillip Gauger, Jianqiang Zhang, Terry Specht, Aaron Gilbertie, William Minton, Ying Fang, Gourapura J. Renukaradhya

**Affiliations:** Food Animal Health Research Program (FAHRP), OARDC, Department of Veterinary Preventive Medicine, The Ohio State University, Wooster, OH 44691 USA; College of Animal Science and Technology, Guangxi University, Nanning, China; Department of Diagnostic Medicine and Pathobiology, College of Veterinary Medicine, Kansas State University, Manhattan, KS 66506 USA; Veterinary Diagnostic and Production Animal Medicine, Iowa State University, Ames, IA USA; Four Star Veterinary Services, Chickasaw, OH 45826 USA

## Abstract

Porcine epidemic diarrhea virus (PEDV) is an economically devastating enteric disease in the swine industry. The virus infects pigs of all ages, but it cause severe clinical disease in neonatal suckling pigs with up to 100% mortality. Currently, available vaccines are not completely effective and feedback methods utilizing PEDV infected material has variable success in preventing reinfection. Comprehensive information on the levels and duration of effector/memory IgA and IgG antibody secreting B cell response in the intestines and lymphoid organs of PEDV-infected sows, and their association with specific antibody levels in clinical samples such as plasma, oral fluid, and feces is important. Therefore, our goal in this study was to quantify PEDV specific IgA and IgG B cell responses in sows at approximately 1 and 6 months post-infection in commercial swine herds, including parity one and higher sows. Our data indicated that evaluation of both PEDV specific IgA and IgG antibody levels in the plasma and oral fluid (but not feces) samples is beneficial in disease diagnosis. PEDV specific B cell response in the intestine and spleen of infected sows decline by 6 months, and this associates with specific antibody levels in the plasma and oral fluid samples; but the virus neutralization titers in plasma remains high beyond 6 months post-infection. In conclusion, in sows infected with PEDV the presence of effector/memory B cell response and strong virus neutralization titers in plasma up to 6 months post-infection, suggests their potential to protect sows from reinfection and provide maternal immunity to neonates, but challenge studies are required to confirm such responses.

## Introduction

Porcine epidemic diarrhea (PED) clinically manifests as severe watery diarrhea with subsequent dehydration in all ages of swine, but highly severe in sucking pigs [1]. Other clinical signs of PED include vomiting and anorexia. PED is characterized by the mortality rate of 30–100% in neonates, and high morbidity but low mortality in weaned pigs [2]. Economic losses due to elevated mortality and decreased production by PEDV are significant in the US swine herds. PED virus (PEDV) is the causative agent of PED. PEDV was detected on multiple US swine farms in April of 2013 [1, 3], and the virus has continued to spread through swine producing states at an alarming rate until the end of 2014. Over 45 779 PEDV tests have been conducted in the US between May 2013 and March 2014, and reported 4757 cases (~10%) positive in 27 states [4].

PEDV is an enveloped virus having 28 kb genome and encode four structural proteins, spike (S), envelope (E), membrane (M), and nucleocapsid (N) [5, 6]. The S protein of PEDV is the principle surface glycoprotein involved in virus attachment and entry, and it contains virus-neutralizing B cell epitopes [7–9].

PEDV continues to infect naive swine farms breaching strict biosecurity protocols for unknown reasons or has re-infected breeding farms after implementing feedback strategies. Piglets are expected to be protected from the clinical disease through colostral immunity received from immune dams [10]. However, protection from infection and shedding has been variable with occasional failure of feedback regimens. Control and prevention of PEDV is one of the major hurdles to the swine industry in the US.

Currently, available vaccines are not completely effective and feedback methods utilizing PEDV infected material has shown varied success in preventing reinfection. This could be attributed to non-availability of reliable diagnostic tools to monitor the protective herd immune status in sows. Moreover, information about levels and duration of PEDV herd immune status in sows is important to implicate appropriate control measures at verge of disease outbreaks. Therefore, it is critical to develop standardized isotype antibody targeted assays to determine the association of clinical samples data with PEDV specific B cell response at the intestines and lymphoid tissues of sows recovered from PED under field conditions. In this study we quantified both PEDV specific IgA and IgG antibody levels in the clinical samples (plasma, oral fluid, and feces) and associated that to the isotype specific B cell responses in the intestine and lymphoid tissues of PED infected sows in commercial breeding herds of two different parities (primiparous and multiparous).

## Materials and methods

### Cells

Vero cells (ATCC^®^ CCL-81) were cultured in Minimum Essential Media (Gibco, CA, USA) supplemented with 10% heat inactivated fetal bovine serum (Atlanta Biologicals, GA, USA), 2 mM l-glutamine (Gibco) and antibiotic/antimycotic solution (HyClone, UT) at 37 °C in a humidified atmosphere with 5% CO_2_. For preparation of virus stocks and in virus neutralizing (VN) assay, the MEM was supplemented with tocylsulfonyl phenylalanyl chloromethyl ketone (TPCK)-trypsin (1 μg/mL) (Sigma, MO, USA), 0.3% tryptose phosphate broth (Sigma), 0.02% yeast extract (BD, MD) and antibiotic/antimycotic solution (HyClone, UT, USA).

### Virus

PEDV strain KS 14-01 was isolated from PED infected field fecal samples at Kansas State University, and was propagated in Vero cells. Confluent cell monolayer was washed with sterile phosphate-buffered saline (PBS) twice before infecting with the virus, and after 1 h of adsorption at 37 °C additional infection medium was added without removing the inoculum. The virus induced cytopathogenic effect was reached to approximately 90% in 2–3 days. The virus culture supernatant was collected after freeze-thawing two times, and then clarified at high speed centrifugation (3000 × *g* for 30 min) and purified the viral antigen by ultracentrifugation through a 20% (wt/vol) sucrose cushion (100 000 × *g* for 2 h). Both the stock virus and viral antigen were stored at −80 °C until used. Viral protein concentration was measured by micro BCA protein assay kit (Thermo Scientific, IL, USA) and virus titer was determined by IFA as described previously [11–13].

### Animals

Three swine breeding farms located in the Middle Eastern part of the Unites States were chosen for this study, with the two farms having the history of piglet mortality and laboratory confirmation of PEDV infection by ELISA at approximately 1 and 6 months before necropsy, and the third farm had no history of PED infection. Six sows each from primiparous and multiparous group with a total of 36 sows with the history of PEDV infection or no infection were used in this study (Table 1). Animals were euthanized in a slaughter plant (Bob Evans slaughterhouse, Xenia, Ohio) according to the standard procedures with necessary efforts to minimize suffering of animals. All the procedures on animals used in this study were approved by the Committee on the Ethics of Animal Experiments of The Ohio State University. On the day of necropsy, plasma, oral swab, and fecal samples were collected for virus specific antibody isotype titration; and ileum, mesenteric lymph nodes (MLN), and spleen samples were collected in MEM containing antibiotics and antifungal for isolating mononuclear cells (MNCs).

**Table 1:** **Grouping of sows at different stages of PEDV infection.**

Groups	No. of sows	PEDV infection	Status	Abbreviation
1	6	Mock	Primiparous	Mock-PP
2	6	Mock	Multiparous	Mock-MP
3	6	1 month post-infection	Primiparous	1 m PI-PP
4	6	1 month post-infection	Multiparous	1 m PI-MP
5	6	6 months post-infection	Primiparous	6 m PI-PP
6	6	6 months post-infection	Multiparous	6 m PI-MP

A total of 36 sows from three different swine breeding farms located in the Mid-Eastern part of the US, having a clear PEDV infection history (uninfected, 1 month or 6 months post-infected) were selected, and six sows each from primiparous and multiparous groups were transported to a slaughter plant in Ohio. Clinical samples such as blood (plasma), oral fluid, and feces, and tissues of ileum, mesenteric lymph nodes, and spleen were collected on the day of necropsy.

### Isolation of immune cells

MNCs from ileum, MLN, and spleen were isolated as previously described [14–17] with a few modifications. Briefly, ileum, spleen, and MLN tissues were cut into tiny pieces and only ileum tissues were treated with Type II collagenase after treating with EDTA and dithiothreitol. Cell suspensions were obtained after passing the digested tissues of ileum, spleen, and MLN through stainless steel Cellectors fitted with an 80 μm mesh screen (Cellector, FL, USA). The harvested MNCs were subjected to density gradient centrifugation with 43% and 70% Percoll, and the cells in the interface were collected and filtered through 40 μm cell strainer (BD Falcon, MA, USA) and re-suspended in enriched-RPMI (E-RPMI, RPMI containing 10% FBS, 200 μm HEPES, 1 mM sodium pyruvate, 25 μm 2-ME, 1x non-essential amino acid, and 1x antibiotic and antifungal). The viability of cells was confirmed by trypan blue dye exclusion method, and counted using a hemocytometer.

### In vitro stimulation of MNCs with PEDV antigen

MNCs isolated from ileum, MLN, and spleen were plated in 24-well cell culture plate (25 × 10^6^ cells/well) in 2 mL E-RPMI in the presence of semi-purified PEDV viral antigen (25 μg/mL), and cells treated with medium alone or lipopolysaccharide (25 μg/mL) were included as controls. Cells were cultured for 6 days at 39 °C with 5% CO_2_, and 0.5 mL of E-RPMI was added to each well on every second day. Supernatants were collected to measure PEDV specific IgA and IgG antibody by ELISA. Cells were harvested, washed using PBS, re-suspended in E-RPMI, counted and used for detecting the population of PEDV-specific IgA and IgG antibody secreting cells (ASC) by enzyme linked immunospot (ELISPOT) assay, and for elucidating the frequency of IgA^+^ and IgG^+^ B cells by flow cytometry.

### Antibody isotype ELISA

PEDV specific IgA and IgG antibody levels were determined as described previously [15, 16]. Briefly, pre-titrated amounts of PEDV whole virus-derived antigen (5 μg/mL), recombinant PEDV S protein (5 μg/mL) or M protein (10 μg/mL) diluted in 50 mM carbonate buffer (pH 9.6) were coated overnight at 4 °C in the 96-well ELISA plates (Corning, MA, USA). Plates were blocked using 10% nonfat milk in PBS containing 0.05% Tween 20. Plasma sample was diluted 1:200, 1:800, 1:3200, and 1:12 800, and oral fluid and fecal samples were diluted 1:8, 1:32, 1:128 and 1:512. The supernatants harvested from stimulated MNCs were diluted 1:2 and 50 μL of each of the samples was applied into duplicate wells and incubated for 1 h at room temperature (RT). Plates were washed and horseradish peroxidase-conjugated goat anti-pig IgA (Bethyl laboratories) or IgG antibody (KPL) (1:5000) was added to the plates and incubated for 1 h. The reaction was developed using TMB peroxidase substrate and stopped using 1 M phosphoric acid and plates were read at OD_450_.

### Virus titration and virus neutralizing (VN) antibody assays

PEDV titer in stocks and VN antibody titer in clinical samples were analyzed by IFA as described previously [11–13] with a few modifications. Briefly, confluent Vero cell monolayers in 96-well plates were washed once using PBS before seeding the samples. For virus titration, tenfold serially diluted virus stock was added to cells plate in quadruplicate; for VN titration, clinical samples were UV treated (254 nm for 45 min) and heat inactivated (56 °C for 30 min), and twofold serially diluted samples were incubated with equal volume of PEDV (50 TCID_50_ per well) for 1.5 h at 37 °C, and the mixture was transferred into Vero cells grown in microtiter plates in duplicate. Plates were incubated for 1 h at 37 °C and infection medium was added after the wells were washed once using PBS and incubated for 24 h at 37 °C. Plates were washed and fixed using acetone-Milli-Q water (8:2) mixture for 10 min at RT, and dried completely before immunostained. Cells were treated with anti-PEDV N protein specific monoclonal antibody (SD6-29) (Medgene lab, SD, USA) (1:1000) for 2 h at 37 °C, followed by treatment with Alexa Fluor 488 conjugated goat anti-mouse IgG (H + L) (Invitrogen, CA, USA) secondary antibody (1:4000). The plate was examined under a fluorescent microscope after mounting with glycerol-PBS (6:4). The virus induced cytopathic effect was examined under a fluorescent microscope, and the titer was calculated using the Reed and Muench method. The viral titer was expressed in 50% tissue culture infective dose (TCID_50_) per mL. The VN titer was determined to be the reciprocal dilution of plasma/oral fluid/fecal samples that induced greater than 90% inhibition of viral infection as shown in virus control wells (Figure 2).

### Enzyme linked immunospot (ELISPOT) assay for quantifying PEDV-specific ASCs

ELISPOT assay was performed to analyze the population of PEDV specific IgA and IgG antibody secreting B cells as described previously [14–17] with few modifications. Briefly, two kinds of PEDV whole virus-derived antigen coated plates were used in this study. (i) Vero cells infected and fixed cell culture plates: PEDV (100 μL of 10^4^ TCID_50_/mL) containing 1 μg/mL of TPCK-trypsin was inoculated to confluent Vero cell monolayers in 96-well tissue culture plate. Cells were fixed with 80% acetone at 16 h post-infection and plates were stored at −20 °C. At that stage about 80% of cells were infected as tested by IFA, and the cell monolayer was still intact. Mock-infected cells treated exactly the same way were used as control. (ii) PEDV whole virus-derived antigen immunocaptured plates: Semi-purified viral antigen (25 μg/mL) diluted in 50 mM carbonate buffer (pH 9.6) was coated overnight at 4 °C in nitrocellulose-based 96-well microtiter plates (Millipore, MA, USA). Plates were washed with PBS and blocked with E-RPMI for 1 h at RT and stimulated MNCs of ileum, MLN, and spleen were plated at three tenfold dilutions in duplicate wells starting from 5 × 10^5^ cells/well. All plates were incubated for 16 h at 39 °C with 5% CO_2_, and washed with PBS containing 0.05% Tween 20 (PBST). The antibody production was detected using horseradish peroxidase-labeled affinity-purified goat anti-pig IgA (Bethyl laboratories, Texas) or IgG (KPL, Maryland) diluted 1:2000 in PBST and incubated for 2 h at 37 °C. The color was developed using 3-amino-9-ethylcarbazole substrate (Sigma-Aldrich, St. Louis, USA) and spots were counted using the AID ELISPOT Reader System (Autoimun Diagnostika GmbH Strassberg, Germany). Data were expressed as the mean numbers of ASCs per 5 × 10^5^ MNCs.

### Flow cytometric analyses

The frequencies of IgA^+^ and IgG^+^ B cells from 100 000 acquired events of immunostained MNCs were determined by flow cytometry as described previously [18] with a few modifications. Briefly, MNCs were stimulated with PEDV whole virus-derived antigen as described above and immunostained with mouse anti-pig IgA mAb (Clone K60 1F1, AbD Serotec) followed by goat anti-mouse IgG1 conjugated to APC/CY7 and rabbit anti-pig IgG conjugated to Texas Red. Subsequently, cells were fixed, permeabilized and then intracellular stained using FITC conjugated rat anti-mouse CD79β antibody (Clone AT107-2, AbD Serotec), which was shown to cross-react with pig B cells [18]. Cells were acquired using BD Aria II flow cytometer and analyzed using the FlowJo software.

### Ethics statement

This study was carried out in strict accordance with the recommendations by Public Health Service Policy, United States Department of Agriculture Regulations, the National Research Council’s Guide for the Care and Use of Laboratory Animals, and the Federation of Animal Science Societies’ Guide for the Care and Use of Agricultural Animals in Agricultural Research and Teaching and all the relevant institutional, state, and federal regulations and policies regarding care and use of animals at the Ohio State.

### Statistical analysis

All data were expressed as the mean ± standard error of mean (SEM) of six sows. Statistical analyses were performed by one-way ANOVA followed by Tukey’s post hoc test (GraphPad InStat 5.0 prism software), and the *P * value of <0.05 was considered significant.

## Results

### Antibody response in PEDV infected sows

**Figure 1:**
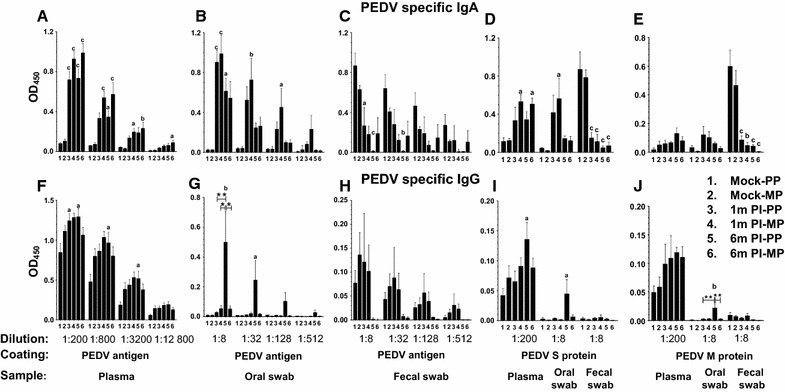
**Quantification of PEDV-specific IgA and IgG antibody titers in clinical samples of PEDV infected sows**. Samples were collected from the uninfected and post PEDV-infected sows as indicated in the Table 1. PEDV specific IgA (**A**–**E**) and IgG (**F**–**J**) antibody titers were quantified by ELISA, with plates coated either using PEDV whole virus-derived antigen (**A**–**C** and **F**–**H**), S protein (**D**, **I**) or M protein (**E**, **J**); in plasma (**A**, **D**–**F, I, J**), oral fluid (**B, D, E, G, I, J**), and fecal (**C**–**E** and **H**–**J**) samples. Each bar represents mean OD_450_ value ± SEM from six sows. Lowercase alphabet indicates statistically significant difference between uninfected and PEDV-infected corresponding parity sow groups, and asterisk indicates statistical difference between the indicated PEDV-infected sow groups (“a” or *P < 0.05, “b” or **P < 0.01 and “c” or ***P < 0.001).

Details of sow groups used in this study are provided in Table 1. Clinically, sows were healthy before transportation to the slaughter plant at Xenia in Ohio and rested for a day before necropsy. PEDV specific IgA and IgG antibody responses were determined in plasma, oral fluid, and fecal samples using PEDV antigen, recombinant PEDV S and M protein coated plates. Our results indicated PEDV specific IgA antibody response in the samples of plasma (Figure 1A) and oral fluid (Figure 1B) from both one and 6 months post-infected sows was significantly higher than uninfected sows, and a similar trend was present in recombinant S (but not M) protein coated plates (Figures 1D and E). Surprisingly, in fecal samples the specific IgA antibody against PEDV whole virus-derived antigen (Figure 1C), S protein (Figure 1D), and M protein (Figure 1E) was absent.

Although PEDV specific IgG antibody levels in the plasma of infected sows was significantly higher than uninfected sows (Figures 1F, I and J), the background optical density values of IgG from uninfected sows was higher compared to respective IgA levels (Figures 1A, D and E). In oral fluid samples of 6 months post PEDV-infected primiparous sows, specific IgG antibody levels against whole viral antigen and recombinant S and M proteins were significantly higher than uninfected and other three infected sow groups (Figures 1G, I and J). PEDV specific IgG antibody in fecal samples was absent in infected sows (Figures 1H, I and J). Our data suggests that for diagnostic antibody analysis of PEDV infection in sows, the plasma and oral fluid samples (but not fecal samples) are reliable and consistent.

**Figure 2:**
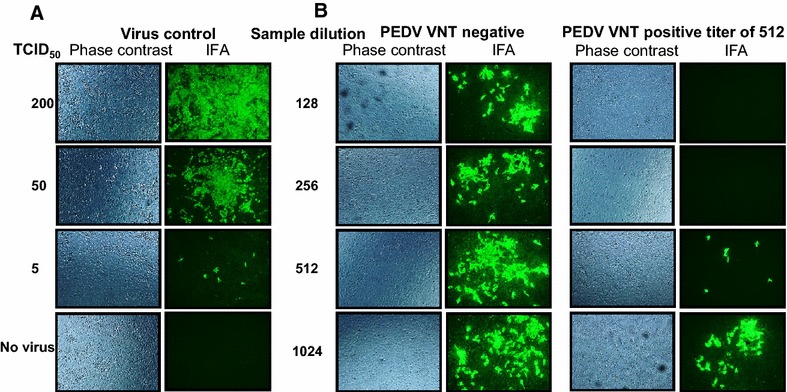
**Representative pictures showing virus neutralizing antibody response in PEDV infected sows plasma samples**. **A** Fifty TCID_50_ of virus was used in the NA assay which showed appreciable quantity of infected cells by immunofluorescence (IFA) assay at 24 h post-infection. **B** Each of representatives experimental plasma sample of PEDV uninfected and infected sows were twofold serially diluted from 1:8 to 1:1024 and mixed with 50 TCID_50_ of PEDV; and Vero cells treated with that mixture were subjected to IFA and examined under 200×magnification. VN titer was expressed as the reciprocal of the highest dilution ratio of test samples that caused greater than 90% reduction in virus induced fluorescence foci units compared to that of virus control.

**Figure 3:**
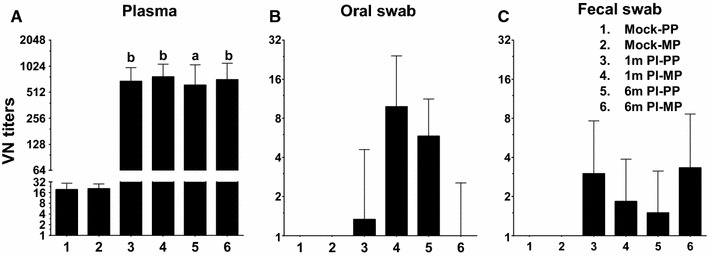
**Virus neutralizing antibody titers in PEDV infected sows**. (**A**) Plasma, (**B**) Oral fluid and (**C**) fecal samples were analyzed for the VN titers against PEDV by immunofluorescence assay. The VN titer was determined as the reciprocal dilution of the test sample that induced greater than 90% inhibition of viral infection. Each bar represents mean VN titers ± SEM from six sows. Lower case alphabet indicates a statistically significant difference (“a” *P* < 0.05 and “b” *P* < 0.01) between mock-uninfected and PEDV-infected corresponding primiparous and multiparous sow groups.

Further, VN antibody titers against PEDV in infected sow samples were quantified by IFA. For this assay, 50 TCID_50_ of PEDV in each well was used, which showed substantial amount of infected cells at 24 h post-infection (Figure 2). Our data revealed that in plasma of PEDV uninfected sows, the background VN titer was up to 16. But, in infected sows, the VN titer was very high in all four infected groups with a titer of 512 (Figure 3A), suggesting that even after 6 months the VN titer in both primiparous and multiparous sows remained high in the plasma. The VN titers in oral fluid and fecal samples were <8 in all the infected sows (Figures 3B and C).

### Flow cytometry analysis of PEDV specific IgA and IgG positive B cells in infected sows

**Figure 4:**
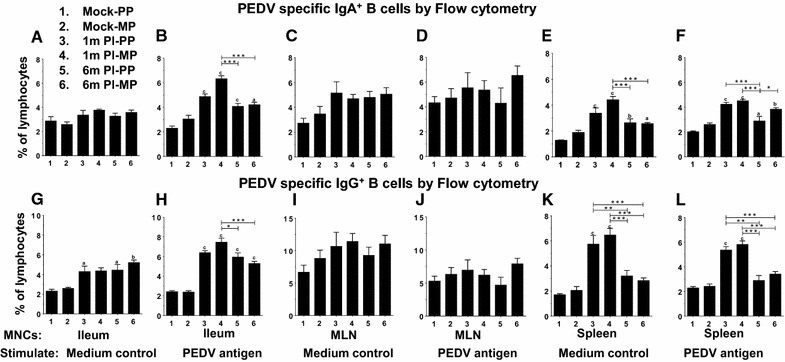
**Flow cytometry analyses to determine the frequency of IgA** ^**+**^ **and IgG** ^**+**^ **B cells in mucosal tissues of PEDV infected sows**. Mononuclear cells were isolated from ileum (**A, B, G, H**), mesenteric lymph nodes (**C, D, I, J**), and spleen (**E, F, K, L**), and stimulated ex vivo with PEDV whole virus-derived antigen (**B, D, F, H, J, L**) or medium control (**A, C, E, G, I, K**) for 6 days. The frequency of B cells positive for IgA (**A**–**F**) and IgG (**G**–**L**) antibodies were determined by flow cytometry. Each bar represents the mean of percent of lymphocytes positive for IgA or IgG positive B cells ± SEM from six sows. Lowercase alphabet indicates statistically significant difference between uninfected and PEDV-infected corresponding parity sow groups, and asterisk indicates statistical difference between the indicated PEDV-infected sow groups (“a” or *P < 0.05, “b” or **P < 0.01 and “c” or ***P < 0.001).

Flow cytometry analysis allows simultaneous multi-parametric analysis of the physical and chemical characteristics of immune cells. MNCs isolated from ileum, MLN, and spleen were stimulated ex vivo using PEDV whole virus-derived antigen for 6 days, and subsequently analyzed the frequency of effector/memory IgA^+^ and IgG^+^ B cells by flow cytometry. Ileum MNCs of PEDV infected sows stimulated with the virus antigen had significantly higher frequencies of both CD79^+^IgA^+^ and CD79^+^IgG^+^ B cells compared to uninfected sows (Figures 4B and H). In addition, the frequency of CD79^+^IgG^+^ B cells (but not CD79^+^IgA^+^ B cells) were also significantly higher in ileum MNCs unstimulated with medium control of three groups of PEDV infected sows (except 1 month multiparous sows) compared to uninfected sows (Figures 4A and G). Specifically, antigen specific CD79^+^IgA^+^ and CD79^+^IgG^+^ B cell populations in the ileum of 1 month post PEDV-infected multiparous sows were significantly higher than 6 months post-infected animals with both primiparous and multiparous status (Figures 4B and H). However, there was no significant changes in the frequency of both CD79^+^IgA^+^ and CD79^+^IgG^+^ B cells in the MLN MNCs of PEDV infected sows, either stimulated with viral antigen (Figures 4D and J) or unstimulated medium control (Figures 4C and I).

In the spleen of all four post-PEDV-infected sow groups the frequency of CD79^+^IgA^+^ B cells was significantly higher compared to their uninfected age-matched counterparts, either unstimulated or stimulated with PEDV whole virus-derived antigen (Figures 4E and F). However, in both primiparous and multiparous sows at 1 month post PEDV-infection, a significantly higher frequency of CD79^+^IgG^+^ B cells was observed compared to both uninfected and 6 months post-infected counterparts, either unstimulated or stimulated with PEDV whole virus-derived antigen (Figures 4K and L). Further, in the spleen of multiparous sows at 1 month post PEDV-infection, increased frequency of CD79^+^IgA^+^ B cells in unstimulated MNCs was significantly higher than that of primiparous and multiparous sows at 6 months post-infection (Figure 4E). In spleen of primiparous sows at 6 months post PEDV-infection, antigen specific CD79^+^IgA^+^ memory B cell population in antigen-stimulated splenocytes was significantly less compared to that in multiparous sows at 1 and 6 month post-infection (Figure 4F). Overall, our data indicated that in PEDV-infected multiparous sows, CD79^+^IgA^+^ B cell response is stronger than that of primiparous infected counterpart.

**Figure 5:**
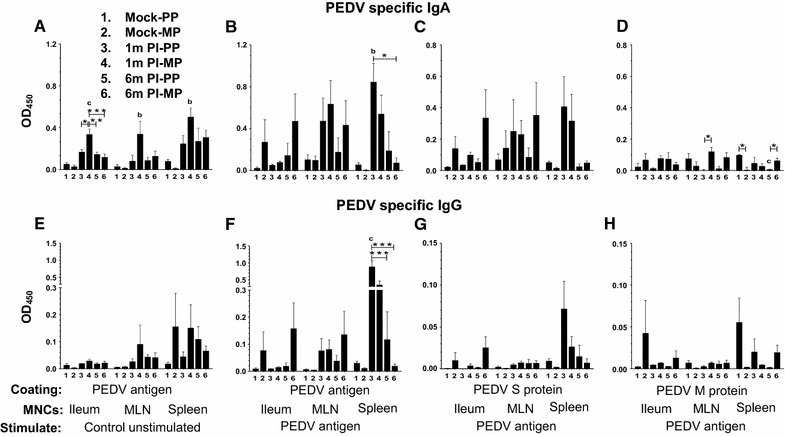
**Analysis of PEDV specific IgA and IgG antibodies secreted by stimulated MNCs of ileum, mesenteric lymph nodes, and spleen**. Culture supernatants were harvested on day six from MNCs stimulated ex vivo with PEDV whole virus-derived antigen (**B**–**D**, **F**–**H**) or control unstimulated (**A, E**). PEDV specific IgA (**A**–**D**) and IgG (**E**–**H**) levels were determined by ELISA, with plates coated either with PEDV whole virus-derived antigen (**A**, **B** and **E**, **F**), S protein (**C, G**) or M protein (**D, H**). Each bar represents mean OD_450_ value ± SEM from six sows. Lowercase alphabet indicates statistically significant difference between uninfected and PEDV-infected corresponding parity sow groups, and asterisk indicates statistical difference between the indicated PEDV-infected sow groups (“a” or *P < 0.05, “b” or **P < 0.01 and “c” or ***P < 0.001).

In the culture supernatants harvested at day six cultures of ileum, MLN, and spleen MNCs from multiparous sows at 1 month post-infection, a significantly increased secretion of PEDV specific IgA antibodies specific to the virus was detected (Figure 5A). While in primiparous PEDV infected sows splenocytes stimulated with antigen, a significantly increased IgA antibody secretion was detected (Figure 5B). While numerically increased (but not significant) trends in IgA antibody production was observed in PEDV infected sows derived MNCs of all three tissues against the S protein, and against M protein the antibody levels were very low (Figures 5C and D). In the splenocytes from primiparous sows at 1 month post PEDV-infection, a significantly higher secretion of virus specific IgG antibodies was detected (Figure 5F).

### Quantification of PEDV secreting IgA and IgG ASC in infected sows

**Figure 6:**
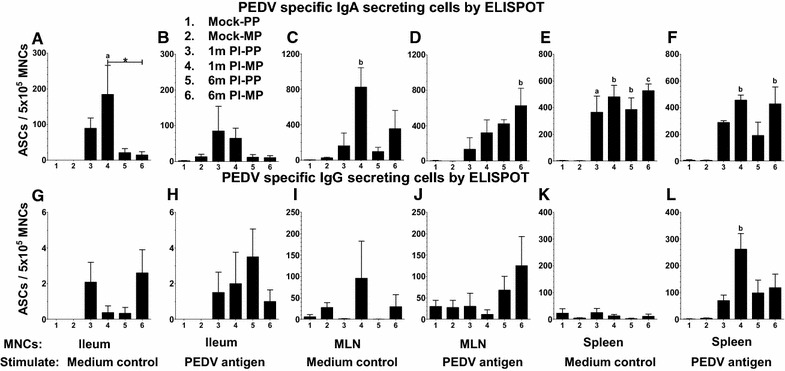
**ELISPOT analyses to quantify PEDV specific IgA and IgG antibody secreting cell population in ileum, mesenteric lymph nodes, and spleen of PEDV infected sows**. MNCs isolated from ileum (**A, B, G, H**), MLN (**C, D, I, J**), and spleen (**E, F, K, L**) were stimulated ex vivo with PEDV whole virus-derived antigen (**B, D, F, H, J, L**) or medium control (**A, C, E, G, I, K**) for 6 days. PEDV specific IgA (**A**–**F**) and IgG (**G**–**L**) antibody secreting cells (ASCs) were detected by ELISPOT. Each bar represents mean number of PEDV-specific ASCs per 5 × 10^5^ MNCs from six sows. Lowercase alphabet indicates statistically significant difference between uninfected and PEDV-infected corresponding parity sow groups, and asterisk indicates statistical difference between the indicated PEDV-infected sow groups (“a” or *P < 0.05, “b” or **P < 0.01 and “c” or ***P < 0.001).

ELISPOT assay allows visualization of the secretory product(s) of individual activated cells, and thus provides both qualitative and quantitative information on T and B cell responses. Population of PEDV specific IgA and IgG secreting cells (ASCs) present in every 0.5 million MNCs of ileum, MLN and spleen were enumerated by ELISPOT assay (Figure 6). To perform this assay, we used both PEDV whole virus-derived antigen containing fixed Vero cells plate and semi-purified PEDV antigen immuno-captured plate. Both the methods detected similar trend in the frequencies of both IgA and IgG ASCs in PEDV infected sow groups, but in fixed Vero cell antigen coated plates, a fivefold to tenfold higher numbers of ASCs were detected compared to the other, therefore fixed cell antigen plate data is shown here (Figure 6). As expected we did not detect PEDV specific IgA and IgG ASCs in both uninfected primiparous and multiparous sows (Figure 6). Overall, the population of PEDV specific IgA ASCs was greater than IgG ASCs in all three tissue derived MNCs (Figure 6).

In post 1 month PEDV-infected multiparous sows ileum MNCs, cultured for 6 days without any further antigenic stimulation, the number of virus-specific IgA ASCs (~200) were significantly greater compared to control uninfected and 6 months post PEDV-infected (10 ASCs) sows (Figure 6A); and a similar trend was present in stimulated ileum MNCs (Figure 6B). In contrast, the number of virus-specific IgG ASCs were too low (<5) in the ileum MNCs (Figures 6G and H). In MLN MNCs, the number of PEDV-specific IgA ASCs in multiparous sows at 1 and 6 months post PEDV-infection were significantly higher compared to uninfected control sows (Figures 6C and D). In unstimulated MNCs of spleen, frequency of PEDV-specific IgA ASCs (~400) in all four PEDV-infected sow groups were significantly higher than that from uninfected control sows (Figure 6E); and only in PEDV-infected multiparous sows, a significant increase in IgA ASCs in viral antigen-stimulated MNCs was observed compared to uninfected animals (Figure 6F). However, significantly greater numbers of PEDV-specific IgG ASCs (~300) were present only in the PEDV whole virus-derived antigen stimulated MNCs from multiparous sows at 1 month post PEDV-infection in comparison to that of uninfected control sows (Figure 6L).

## Discussion

We evaluated PEDV specific B cell response in 24 infected sows at approximate 1 and 6 months post-infection, comprising of both primiparous and multiparous sow groups and included 12 age-matched control uninfected sows. In an earlier study, PEDV whole virus antigen based ELISA was developed using the virus grown in Vero cells, and the viral antigen used in the assay was extracted using 0.2% Triton X-100 to analyze antibodies in 1024 field samples at the herd level [19]. Though we followed a similar method of growing the virus, but the ELISA antigen was extracted from freeze-thawed viral culture fluid subjected to 20% sucrose cushion ultracentrifugation, probably the cell debris still present in our antigen preparation would have contributed to the observed high background activity in IgG ELISA in plasma, which needs further investigation.

PEDV S protein is a class I viral fusion protein [20], and is cleaved by host-derived proteases when virus enters the susceptible cell [21]. The S protein contains B cell epitopes for induction of neutralizing antibodies [22]. The S protein-based ELISA was found to be sensitive to evaluate antibody response against PEDV in plasma and colostrum samples [23–26]. In our study, in addition to whole virus antigen, both recombinant PEDV S and M protein based IgA and IgG ELISA was used to estimate the levels of antibody in plasma, oral fluid, and fecal samples of sows; and detected high levels of specific IgA response in infected sows’ plasma followed by in oral fluid, but not in fecal samples. This suggests that PEDV specific antibodies in the fecal samples of infected sows disappear early (approximately 1–2 months) post-infection; in spite of the presence PEDV specific IgA and IgG ASCs in intestines and lymphoid organs at 6 months post-infection. We also noticed that PEDV specific IgA response in plasma (but not in oral fluid) remained high until 6 months. Furthermore, though we found comparable reactivity of PEDV whole virus derived antigen and S protein for detection of specific IgA antibodies, the antibody levels were twofold less in the S protein based ELISA (ODs at 1:200 dilution). Overall, our data suggested that for diagnosis purpose it is ideal to use both whole virus and S protein based IgA and IgG ELISA in herd oral fluid as well as in statistically acceptable number of plasma samples.

Consistent with the results of others [14, 27, 28], we did not detect PEDV specific response in ileum MNCs of uninfected sows, confirming their PEDV negative status and suggesting the need of in vivo priming of the immune system by the live virus to observe the response. But like earlier results in PEDV infected experimental piglets [16], we observed significantly increased population of PEDV specific ASCs and IgA and IgG positive B cells in the ileum and spleen of sows in in vitro cultured MNCs for 6 days in the absence of viral antigen restimulation, indicating the presence of virus specific effector B cells for up to 6 months in PEDV infected sows. In one of our previous study in pigs vaccinated/infected with porcine reproductive and respiratory syndrome virus a similar recall lymphocyte response in lung MNCs and PBMC cultured in vitro in the absence of the virus antigen was observed [12, 29].

Interestingly, we detected substantially higher frequency of PEDV specific ASCs compared to the results of an earlier study [16], this could be attributed to difference in the age of pigs and the virus strain used in the study. At this stage due to lack of pig specific B cell memory marker reagent we could not analyze the frequency of memory B cell pool by flow cytometry in PEDV infected sows.

When PEDV specific IgA and IgG responses in clinical samples were compared with the antibody secreting B cell population in the ileum, MLN, and spleen of sows analyzed by both flow cytometry and ELISPOT assays; surprisingly, multiparous sows had increased humoral response both in terms of antibody and B cell response compared to primiparous animals, and that response was high in 1 month post-infected sows and declined by 6 months. But the VN titers in plasma remained high in all the PEDV-infected sows, suggesting that infected sows could be resistant to reinfection beyond 6 months. However, the level of protection varies depending on extent of viral antigenic diversity, health status of sows, and secondary microbial infections. Due to lack of virus isolation from PEDV-infected sows, the genetic variability between the virus that infected sows and the virus used for ex vivo stimulation could not be analyzed, and thus we could not determine the levels of cross-protection to PEDV in sows.

In an earlier experimental study, 11 days old conventional pigs were infected with PEDV, and specific IgA and IgG ASCs in the intestines were detected at higher levels than in systemic sites (spleen and PBMC) [16]. However, in sows naturally infected with PEDV, we observed higher levels of IgA and IgG ASCs in spleen than in intestines, suggesting that systemic response is equally important and it persists for longer time in infected sows compared to piglets. Suckling and gnotobiotic pigs infected with TGEV and rotavirus, respectively, had higher numbers of specific IgG ASCs compared to IgA ASCs in the intestines [14, 16, 27, 30]. But in our study, in both one and 6 months post PEDV-infected primiparous and multiparous sows, the frequency of specific IgA ASCs were greater than IgG ASCs in the intestines, MLN, and spleen. This suggests the enhanced susceptibility of neonatal pigs to PEDV is responsible for induction of greater levels of IgG ASCs in the intestinal tissues in comparison to that of sows which experience low to mild level of PEDV-infection.

Since PEDV infection is localized to intestinal mucosa, stimulation of systemic immune response especially in piglets is at very low levels [31, 32]. However, our study in sows suggested that systemic response in spleen is predominant over the response in intestines; the differences might be due to time frame of sampling and the age of the animals. Immunologically, the possible reason could be the migration of activated PEDV antigen-bearing dendritic cells to spleen, resulting in activation of naïve B and T cells. Alternatively, the memory lymphocytes from the mucosa- associated lymphoid tissues could migrate to spleen and bone marrow [33]. In summary, PEDV specific IgA and IgG B cells in infected sows were detectable at higher levels in ileum and spleen compared to that in MLN using both ELISPOT (total B cell response) and flow cytometry (detection of frequency of specific IgA and IgG B cell response) assays, with the later assay demonstrating a more robust result compared to the former in detecting antigen and isotype specific B cells. However, detection of antibody isotype specific B cell response with a memory phenotype marker in mucosal tissues will be highly beneficial to determine PEDV vaccine or infection induced memory in pigs.

In conclusion, our results suggests that detection of PEDV specific IgA in plasma and oral fluid samples has a potential diagnostic implication, and estimation of VN titers in plasma could serve to determine the protective immune status. Although PEDV specific B cell response decline by 6 months in both ileum and spleen of naturally infected sows, the presence of high levels of VN titers suggest that such sows may protect their offspring from PEDV infection by genetically closely related virus by up to 6 months.
